# Unmixing of fluorescence spectra to resolve quantitative time-series measurements of gene expression in plate readers

**DOI:** 10.1186/1472-6750-14-11

**Published:** 2014-02-03

**Authors:** Catherine A Lichten, Rachel White, Ivan BN Clark, Peter S Swain

**Affiliations:** 1 SynthSys, University of Edinburgh, Mayfield Road, Edinburgh, UK; 2 Department of Physiology, McGill University, Promenade Sir William Osler, Montreal, Canada; 3 Biological Sciences, University of Edinburgh, Mayfield Road, Edinburgh, UK

**Keywords:** Gene expression, Fluorescence, Plate readers, Spectral unmixing, Budding yeast, High throughput measurements, Systems biology

## Abstract

**Background:**

To connect gene expression with cellular physiology, we need to follow levels of proteins over time. Experiments typically use variants of Green Fluorescent Protein (GFP), and time-series measurements require specialist expertise if single cells are to be followed. Fluorescence plate readers, however, a standard in many laboratories, can in principle provide similar data, albeit at a mean, population level. Nevertheless, extracting the average fluorescence per cell is challenging because autofluorescence can be substantial.

**Results:**

Here we propose a general method for correcting plate reader measurements of fluorescent proteins that uses spectral unmixing and determines both the fluorescence per cell and the errors on that fluorescence. Combined with strain collections, such as the GFP fusion collection for budding yeast, our methodology allows quantitative measurements of protein levels of up to hundreds of genes and therefore provides complementary data to high throughput studies of transcription. We illustrate the method by following the induction of the *GAL* genes in *Saccharomyces cerevisiae* for over 20 hours in different sugars and argue that the order of appearance of the Leloir enzymes may be to reduce build-up of the toxic intermediate galactose-1-phosphate. Further, we quantify protein levels of over 40 genes, again over 20 hours, after cells experience a change in carbon source (from glycerol to glucose).

**Conclusions:**

Our methodology is sensitive, scalable, and should be applicable to other organisms. By allowing quantitative measurements on a per cell basis over tens of hours and over hundreds of genes, it should increase our understanding of the dynamic changes that drive cellular behaviour.

## Background

Most organisms live in changing environments, and gene and protein networks respond dynamically to extracellular change. Understanding these dynamic responses is one of the challenges of biology [1], but it is difficult to monitor changes within cells as they happen. Microscopy [2], particularly when combined with microfluidics [3], offers a way forward, but requires specialized expertise and equipment.

Most laboratories do, however, have access to a fluorescence plate reader. Fluorescence plate readers allow multiple experiments to be run in parallel on populations of cells in a temperature-controlled environment with optional agitation. Such experiments produce quantitative time-course data measuring absorbance and fluorescence. These data provide information about cell growth and, by using genetically encoded fluorescent reporters, gene expression. Plate readers report only on the mean response of the population of cells and information on the variation around that mean is lost. Nevertheless, such mean data is often suitable for many applications either in itself [4,5] or as additional data to support experiments at the single-cell level.

There are two factors that complicate the analysis of data from plate readers. First, when measuring absorbance, optical density (OD) does not always increase linearly with the density of cells, although this effect can be corrected with a calibration procedure [6,7]. Second, the measured fluorescence, in addition to containing emissions from the fluorescent proteins under observation, also includes contamination from other sources: autofluorescence of the plate and media and autofluorescence from the cells. For weakly expressed proteins, this autofluorescence can dominate the signal from the fluorescent reporter.

Developing an effective technique for separating out the signal of interest from the background autofluorescence would make it possible to collect quantitative data on the mean level of gene expression per cell over time. While various methods have been developed for *Escherichia coli* and applied to fusions of promoters and Green Fluorescent Protein (GFP) [4,8,9], the few published examples of measuring protein expression using a fluorescence reader in other organisms such as yeast have been limited to highly expressed proteins (for instance [10]).

**Figure 1:**
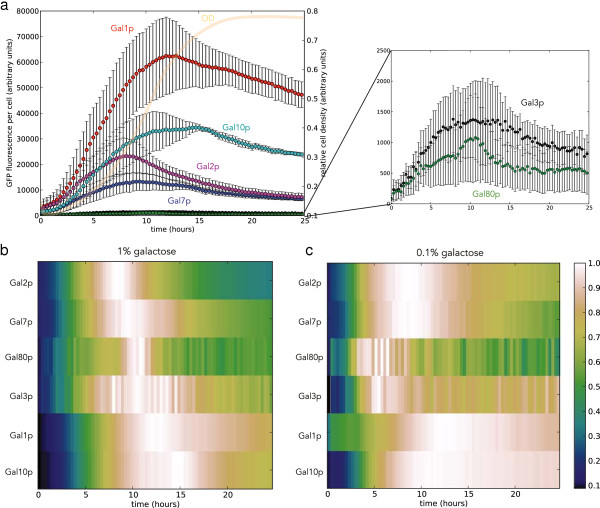
**Spectral unmixing reveals the timing of expression of the GAL genes.** Cells were grown in 2% raffinose, transferred into galactose and followed in the plate reader. **a)** Left-hand axis: Time-series data for mean fluorescence per cell for the Leloir enzymes Gal1, Gal7, and Gal10 and for the galactose permease, Gal2, and the regulators Gal3 (an intracellular sensor of galactose) and Gal80 (a repressor of GAL expression) in 1% galactose. Right-hand axis: Relative cell density (orange). **b)** The same data in **a**, but with the time-series of expression for each gene normalized by its maximum value. The normalized level of expression is indicated by the colour bar. **c)** A similar plot to **b** but for GAL expression in 0.1% galactose. Derivation of the error bars shown is given in Methods.

**Figure 2:**
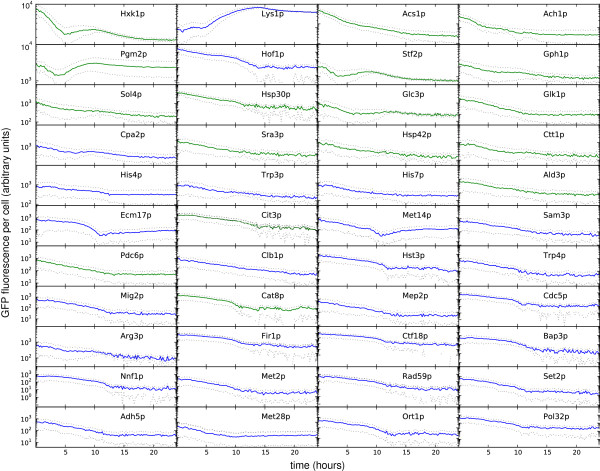
**Spectral unmixing allows dynamic, medium throughput quantification of levels of proteins.** Cells were grown in 3% glycerol, transferred into media with 3% glycerol and 2% glucose, and followed in the plate reader with data taken every 12 minutes. Genes are ordered by maximum level of expression with this maximum decreasing across the rows and down each column (Hxk1p has the highest maximum expression; Pol32p has the lowest maximum expression). Fluorescence values are in arbitrary units, with long tick marks showing a change in fluorescence of a factor of 10. The *y*-axes in the first two rows ranges from 10^3^ to 10^4^ units. The blue curves are protein levels per cell for genes with increased mRNA expression from 0-1 hours, using the data from Wang *et al.*[19]; the green curves are protein levels per cell for genes with decreased mRNA expression from 0-1 hours. Cell density initially increases and then plateaus at approximately 12 hours. Error bars are shown with a dashed line (Methods).

In the following, we describe a technique for quantifying plate reader experiments using spectral unmixing and show that we can capture and quantify a wide range of levels of protein expression in the budding yeast *Saccharomyces cerevisiae*. Budding yeast, one of the world’s most well-studied organisms, has long been a focus for high throughput experiments. We demonstrate that our method is robust enough to allow medium throughput measurements of protein levels in yeast using, for example, the GFP fusion collection [11]. It therefore provides an additional type of data to complement other high throughput characterizations of gene expression, which typically consider only transcription. We illustrate the methodology with two examples: the expression of genes for galactose import and metabolism (Figure 1), a classic example of a eukaryotic response to an environmental change [12], and the response to a shift from a poor to a rich carbon source, where we study expression of 44 genes from the GFP fusion collection (Figure 2).

## Results and discussion

### Correcting autofluorescence following a procedure for bacteria is not appropriate for budding yeast

For experiments with *Escherchia coli*, the fluorescence of interest is often corrected using the autofluorescence of wild-type cells as an estimate for the autofluorescence of cells expressing fluorescently tagged proteins [4,8,9]. For example, to correct for autofluorescence at a particular time point, either the fluorescence per cell for the wild-type cells (the fluorescence at that time point divided by the OD at the same time point) was subtracted from the fluorescence per cell for tagged strains [8] or the fluorescence levels from the wild-type strain interpolated to the OD of the tagged strain was subtracted from the fluorescence of the tagged strains [9].

**Figure 3:**
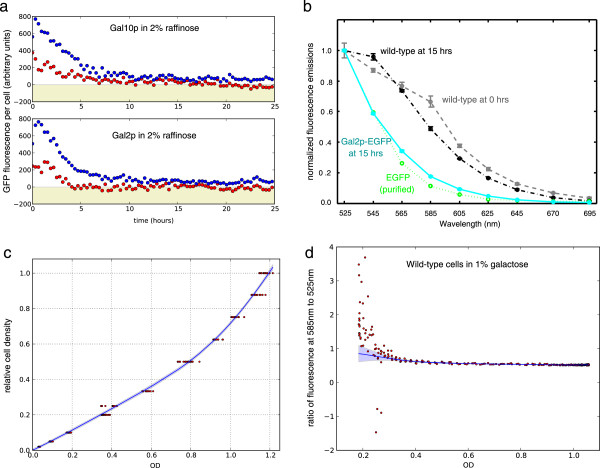
**Estimating the mean fluorescence per cell requires several preliminary steps. a)** Correcting autofluorescence using the wild-type fluorescence per cell, following the method of de Jong *et al.*[8] developed for bacteria, can lead to negative estimates in yeast. Correction using the bacterial method is shown in red; correction using spectral unmixing is shown in blue. Data are for cells grown in 2% raffinose and then grown further in 2% raffinose in the plate reader. Raffinose does not induce *GAL* expression. **b)** Emissions spectra for autofluorescence and enhanced GFP (EGFP) following excitation at 485 nm. Cells were grown in 2% raffinose and then in 2% galactose for the time indicated. Data for purified EGFP were obtained from the website of the R. Tsien laboratory at U.C. San Diego. **c)** The OD correction curve: relative cell density obtained from diluting a culture by known factors plotted against the measured OD of the diluted cultures. The blue line shows a regression and the shading gives 95% confidence limits (plus and minus twice the standard deviation of the posterior distribution found using a Gaussian process). **d)** Estimating *r*_*a*_: the ratio of fluorescence emitted at 585 nm to 525 nm plotted versus OD. The blue line shows a regression and the shading gives 95% confidence limits (found using a Gaussian process). Cells were grown in 2% raffinose and transferred to 1% galactose in the plate reader.

Such correction procedures, however, require that both the tagged and wild-type strains have identical levels of autofluorescence. Typically, though, there are differences in growth curves between strains because of biological reproducibility, perturbations generated by the fluorescent reporter, or both. Such differences are important because autofluorescence need not only depend on the size and stage of growth of the population, but also on the contents of the growth media, which changes over time as nutrients are depleted by the cells and products excreted. Indeed, examining the autofluorescence of wild-type cells revealed that the autofluorescence does vary with the type and amount of sugar available, at least for budding yeast. Further, for low levels of gene expression, strains with fluorescently tagged proteins could have lower fluorescence values than wild-type cells, leading to predictions, with the correction procedure of [8], of negative levels of fluorescence per cell (Methods: Figure 3a). Ideally then, we would like to correct each cell by its own autofluorescence rather than by an average value calculated from other cells in a different well in the plate reader.

### Spectral unmixing corrects autofluorescence

By measuring emissions at two wavelengths, rather than the usual one, we can infer the mean autofluorescence of a particular population of cells in a given well at a given time and so partly meet this goal. We use a single excitation wavelength, but measure emissions both at the wavelength appropriate to the fluorescent reporter and at a higher wavelength where most emissions come from autofluorescence. For example, the emission spectrum of autofluorescence is broader than that of enhanced GFP (Methods: Figure 3b), and we can infer the extent of autofluorescence emissions in the wavelength used for the fluorescent reporter from the autofluorescence emissions measured at the high wavelength.

We use spectral or linear unmixing to remove autofluorescence [13,14]. In linear unmixing, signals measured at each wavelength are assumed to be a linear combination of the different components in the sample. For the plate reader data, we assume that each measured fluorescence is a linear sum of autofluorescence and the fluorescence from any fluorescent reporter present in the cells. At each time point, we make two fluorescence measurements: excitation at 485 nm (near the excitation peak of GFP) and emission at both 525 nm (near the emissions peak of GFP) and at 585 nm (where GFP emissions are much reduced but autofluorescence is still substantial: Figure 3b). For all fluorescence measurements, we first remove the autofluorescence of the media and plasticware by subtracting the fluorescence of wells containing only media.

For our analysis, we assume that: (i) the ratio of emissions of autofluorescence at 585 nm to 525 nm after excitation at 485 nm is the same for the wild-type and tagged strains at a given OD, and (ii) that the autofluorescence and the fluorescence of the tagged strain combine linearly. Our assumption (i) is weaker than assumptions of equal growth curves and equal levels of autofluorescence per cell required by the bacterial correction method. Assumption (ii), as mentioned, is standard (see, for example, [15]).

For each time point, we have four measurements of fluorescence: two for fluorescently tagged strains and two for wild-type strains. The two measurements for the tagged cells are:

(1) $$\begin{array}{l} f_{525}=g+a\\ f_{585}=r_{g}g+r_{a}a \end{array}$$

where *f*_525_ is the fluorescence measured at 525 nm and *f*_585_ is the fluorescence measured at 585 nm. The symbol *g* denotes fluorescence from the protein tags with excitation at 485 nm and measurement at 525 nm, and *a* denotes autofluorescence also from excitation at 485 nm and measurement at 525 nm. We wish to determine *g*. For emissions at 585 nm, we write the fluorescence from the tagged proteins as a product of a constant *r*_*g*_ and *g* and the autofluorescence as the product of *r*_*a*_ and *a*. The constant *r*_*g*_ is the ratio of enhanced GFP emissions at 585 nm to that at 525 nm. Normalized emission of enhanced GFP at 525 nm is 0.570 and normalized emission at 585 nm is 0.065 implying that *r*_*g*_≃0.114 (data from R. Tsien laboratory at U.C. San Diego). This number is consistent with the same fluorescence ratio measured from cells strongly expressing an EGFP-tagged protein (Methods: Figure 3b). The two wild-type measurements only have contributions from autofluorescence:

(2) $$\begin{array}{l} f_{525}^{\text{WT}}=a^{\text{WT}}\\ f_{585}^{\text{WT}}=r_{a}a^{\text{WT}} \end{array}$$

where *r*_*a*_, the ratio of the autofluorescence at 585 nm to that at 525 nm, is assumed to be the same for both wild-type and tagged cells (assumption (i) above), but the autofluorescence of the wild-type cells, *a*^WT^, can be different from the autofluorescence of the tagged cells, *a*.

In essence, our method is then to estimate *r*_*a*_ from the wild-type data, Eqs. 2, as

(3) $$r_{a}=\frac{f_{585}^{\text{WT}}}{f_{525}^{\text{WT}}},$$

and then solve Eqs.1 for *g*, the signal of interest:

(4) $$g=\frac{r_{a}f_{525}-f_{585}}{r_{a}-r_{g}}$$

where the *r*_*a*_ is calculated for wild-type data at the same OD as the OD of the tagged strain. Dividing *g* by OD then gives an estimate of the fluorescence per cell.

In practice, the data are often noisy (Figure 3d) and we allow *r*_*a*_ to change smoothly with OD. Further, we often wish to calculate error bars on our estimates of fluorescence, *g*, and so need to determine how errors in estimating *r*_*a*_ propagate through to the estimation of *g* (Methods). Finally, we present the fluorescence per cell by dividing *g* by the appropriate relative cell density, which we find by correcting OD levels for any non-linear dependence on absolute cell density (Methods).

### Time-series measurements for expression of *GAL* genes

Applying spectral unmixing to measurements of six proteins in the *GAL* pathway tagged with enhanced GFP reveals the timing of *GAL* gene expression (Figure 1). Cells were grown in 2% raffinose and then transferred to galactose and followed in the plate reader. The three Leloir enzymes, Gal1p, Gal7p, and Gal10p, are strongly expressed in response to galactose and convert galactose into a form of glucose [12]. Other *GAL* proteins regulate the response: Gal2p is a galactose permease; Gal3p is a sensor of intracellular galactose and its activation promotes induction of the *GAL* genes; and Gal80p is a transcriptional repressor and (indirectly) reduces *GAL* expression. Both *GAL3* and *GAL80* are only weakly induced, but their expression can still be detected (Figure 1a Inset).

We see (Figure 1b and c) that levels of Gal7p reach their maximum before levels of Gal1p and Gal10p, the other Leloir enzymes, and the ability of Gal7p to remove a toxic intermediate may explain this early expression. Galactose is converted to galactose-1-phosphate, glucose-1-phosphate, and then glucose-6-phosphate before being able to enter glycolysis [12]. This process must be efficient because galactose-1-phosphate is toxic to cells [16]. Gal7p is a uridyl transferase that converts UDP(uridine diphosphate)-glucose and galactose-1-phosphate to UDP-galactose and glucose-1-phosphate. It thus removes toxic galactose-1-phosphate, which is created by the combined action of Gal1p and Gal10p. Having sufficient quantities of Gal7p ready early may therefore prevent a deleterious build up of toxicity.

Excluding the regulatory proteins, Gal3p and Gal80p, the permease Gal2p is expressed first, which may ensure that cells take advantage of the new carbon source as quickly as possible. Both negative feedback through expression of the repressor Gal80p and positive feedback through expression of the sensor Gal3p initiate at similar times and levels of both proteins peak approximately simultaneously. Naively, Gal3p expression might have been expected to precede expression of Gal80p to ensure that the system is initially dominated by positive feedback generating a fast response. The strength of feedback, however, is determined not just by levels of proteins but also by binding affinities, and therefore positive feedback through Gal3p could still dominate expression at the promoters of the Leloir enzymes. Interestingly, both regulatory proteins peak earlier for lower concentrations of galactose (although levels of measurement noise are high).

### Probing gene expression using the collection of GFP fusion proteins

Our method is sufficiently robust that we can simultaneously assay at least tens of genes. A collection of over 4,000 strains of budding yeast has been created [11], each of which expresses a different GFP fusion protein and that potentially allows for high throughput studies of gene expression. Surprisingly the collection has been rarely used in this way, and then only for tens of genes (for instance [17]) or with custom-built microfluidics [18] and never apparently with plate readers. We now show that our methodology combined with robotics could straightforwardly allow expression of hundreds of genes to be followed over time.

After growing 45 strains from the GFP collection in 3% glycerol, we transferred each strain into 2% glucose and followed expression in the plate reader (Figure 2). An increased availability of glucose to starved cells of budding yeast is known to cause substantial transcriptional reprogramming [19]. We had two replicates of each strain, which with two further wells containing wild-type strains and four containing media, filled a 96-well plate. One strain (*SHE1-GFP*) did not grow, but all other genes were amenable to our analysis.

A similar experiment has been performed by Wang *et al.*[19], but with gene expression followed by microarrays up to one hour after the cells were placed in glucose. We used this data to select our strains: those with the largest positive or negative changes in mRNA levels relative to their median level and that were in the GFP collection. To quantify the change in mRNA levels, we used the difference between levels at 0 and 60 minutes after exposure to glucose normalized by the median level of mRNA for that gene (measurements were taken at 0, 20, 40, and 60 minutes).

In general, this measure of change in mRNA based on the microarray data was a poor predictor of the dynamics of the corresponding protein (Figure 2), in agreement with conclusions drawn from proteomics [20]. Although all 17 genes that had decreasing levels of mRNA also had decreasing levels of protein (except perhaps Pgm2p – row 2 & column 1), only one out of the 27 genes with increasing levels of mRNA had increasing levels of protein (Lys1p – row 1 & column 2): the vast majority of genes had falling levels of protein. We emphasize though that our experiment followed cells for almost 24 hours whereas the microarray experiment ran for 1 hour and that the cells stopped growing after about 12 hours when the cell density, as measured by OD, flattened. Reflecting this loss of growth, expression from many genes levels off around 12 hours (Figure 2).

Genes with decreasing levels of mRNA do typically have high levels of proteins, at least initially (they mostly appear in the top half of Figure 2), indicating that these proteins are potentially required for growth on glycerol but not for growth on glucose. Indeed, the hexokinases, Hxk1p (row 1 & column 1) and Glk1p (row 3 & column 4) are induced by non-fermentable carbon sources and repressed in glucose [21]. Further, Acs1p (row 1 & column 3), an acetyl-coenzyme A synthetase, Ach1p (row 1 & column 4), an acetyl coenzyme A hydrolase, Ald3p (row 5 & column 4), an aldehyde dehydrogenase, and Pdc6p (row 7 & column 1), a pyruvate decarboxylase, have been reported to have their expression repressed in glucose [22-25], as has Cat8p (row 8 & column 2), an activator of a variety of genes under non-fermentative growth [26].

Where expression has been investigated, our data largely support previous studies. They are consistent with the expectations for levels of Pgm2p (row 2 & column 1), phosphoglucomutase, which should rise as glucose falls [27], for Hsp30p (row 3 & column 2), a heat shock protein present in stationary phase [28], and for Glc3p (row 3 & column 3), glycogen branching enzyme, whose transcription peaks when glucose is exhausted (at around 12 hours) [29]. Nevertheless, we do not see evidence of the expected induction during late exponential phase of *GPH1* (row 2 & column 4) [30], a glycogen phosphorylase.

## Conclusions

We have introduced a new method to quantify protein expression that uses plate reader measurements of emissions at two wavelengths and spectral unmixing to correct for autofluorescence. Our approach uses linear unmixing and is similar in spirit to others that have been proposed to solve the problem of autofluorescence during flow cytometry [31,32]. While we have considered just one fluorophore, the methodology could readily be applied to samples with two or more [13].

Our method allows medium throughput measurements of gene expression through resources, such as the collection of GFP-protein fusions in budding yeast [11]. Protein levels from the expression of hundreds of genes can be followed over tens of hours. Although other approaches have been applied to bacteria [4,8,9], these methods require that the growth curves of a wild-type and all tagged strains are identical and consequently do not scale to hundreds of strains with different tagged genes, which will not all have the same growth curves. The work in bacteria, though, used GFP-promoter fusions rather than GFP-protein fusions, and promoter fusions may perturb growth rates less (although biological reproducibility will still be an issue).

Time-series data describing the mean behaviour of a typical cell in population of cells is ideal for testing mathematical models of cellular behaviour based on ordinary differential equations [5], and our analysis allows such data to be generated from plate readers. Further, and unlike other methods, we provide error bars on the fluorescence per cell that combine both measurement error and the errors generated by correcting for autofluorescence. Such error bars are invaluable when fitting models to data [33,34].

High throughput studies of gene expression typically focus on levels of mRNA, but levels of mRNA need not correlate with levels of protein [20]. Our methodology combined with strain collections such as the GFP fusion collection will allow complementary data at the level of proteins to be gathered using standard laboratory equipment. This data will increase our understanding of physiological change and cellular decision-making because it is largely proteins, not mRNA, that enact such responses.

## Methods

### Reagents

Low fluorescence synthetic complete media (SC) was made following Sheff *et al.*[35]. We determined that the primary source of media fluorescence was the ammonium salts solution and that the media’s fluorescence increased significantly after both autoclaving and a few days of storage at room temperature. To reduce media fluorescence we therefore used filter sterilisation instead of autoclaving, stored the ammonium salts solution at -20°C, made smaller batches of media to minimise the storage time, and stored the media at 4°C. Sugar solutions were made up from D_(+)_Raffinose pentahydrate, D_(+)_Glucose, and D_(+)_Galactose (Sigma-Aldrich, Steinheim, Germany).

We used black optical bottom 96-well plates (cat. no. 265301, ThermoScientific/Nunc, Rochester, New York, USA), in which we found reduced autofluorescence compared with clear plates. For sterilisation, they were placed in a UV cross linker on high for 2 min (1 min face up, 1 min face down). Film covers were Polyolefin Sealing Tape (cat. no. 235307 Nunc), which contributed less autofluorescence than clear polystyrene lids and reduced evaporation.

### Yeast strains

The strain used was haploid *a* BY4741 *S. cerevisiae* (obtained from EUROSCARF, Frankfurt). To construct strains expressing *GAL* protein-EGFP fusions, we followed the procedure described by Janke *et al.*[36]. The *pYM28* plasmid with an EGFP tag and marker HIS3MX6 (accession number P30240, EUROSCARF toolbox) [37] was amplified with primers containing 40-50 bp of the region just upstream of the target gene’s stop codon, followed by CGTACGCTGCAGGTCGAC (forward primer), and 40-50bp of the reverse strand for the region just beyond the target gene’s coding sequence, followed by ATCGATGAATTCGAGCTCG (reverse primer). PCR products were verified with gel electrophoresis. Then, following PCR product purification with a Qiaquick PCR purification kit (Qiagen), yeast transformation was carried out following a modified LiOAc protocol [37]. For growth and selection, yeast were spread on SC plates containing 2% glucose lacking histidine and incubated for 2-3 days at 30°C. Positive colonies were purified by streaking onto fresh plates. Cultures were stored at -80°C in media containing 10% glycerol.

### Preparing galactose induction experiments

Cells were grown overnight (17-19 hours) in 5 mL SC with 2% raffinose in glass test tubes at 30°C with shaking at 200-230 rpm. In the morning, cells were diluted 10 × into fresh SC with 2% raffinose and incubated further at 30°C with shaking at 200-230 rpm. After 6-8 hours, 180 *μ*L of each sample was placed in wells of a 96 well plate to measure the OD. Then, cells were transferred to 15 mL plastic tubes and washed: they were spun (5 min at 3000 rpm = 1811 RCF) to form a pellet, supernatant was discarded, pellet was resuspended in 11 mL sterile water, and then cells were spun again (5 min at 3000 rpm).

While cells were being washed, the OD was measured and dilutions were calculated to obtain starting OD values for the experiment of 0.25 (wild-type) or 0.3 (cells with enhanced GFP tag). Wild-type cells started at a lower OD to ensure that autofluorescence corrections are possible from the beginning of the induction period. When washing was complete, cells were resuspended in the volume of low fluorescence SC media indicated by the dilution calculations. OD was measured again and adjustments made as necessary to achieve desired starting OD values.

Finally, 180 *μ*L aliquots of each sample were then pipetted into wells of a 96-well plate. 20 *μ*L of the appropriate sterile sugar solutions in water were then added to the wells. A polyolefin film cover was used to cover the plate, and plate reader measurements were begun immediately.

### Measuring using the plate reader

Plate readers were from the Tecan Infinity M200 series (for combined OD and dual-wavelength fluorescence measurements) (Tecan Group Ltd., Switzerland). We set the temperature at 29.9°C (range 29.4°-30.4°C) with linear shaking (6 mm amplitude at 200-220 rpm). For OD measurements, the absorbance wavelength was 595 nm and the measurement bandwidth was 9 nm with 15 reads and 0 ms settle time. For fluorescence measurements, the excitation wavelength was 485 nm, the excitation bandwidth was 9 nm, the emission wavelength was 525 nm (or 585 nm), the emission bandwidth was 20 nm, and reading mode was top with 0 *μ*s lag time, 20 *μ*s integration time, 10 reads, and 0 ms settle time. The gain was set manually to 105. To minimise photobleaching and fluorescence-induced toxicity, measurements were made no more often than every 10 minutes. The plate reader was switched on and adjusted to the correct temperature around 1-2 hours prior to the experiment.

### Medium throughput measurements of gene expression

Selected strains from the yeast GFP fusion collection (Life Technologies, California) were cultured in 100 *μ*l SC with 3% glycerol (w/v) in a 96 well plate at 30°C for 16 hours with shaking. A Biomek FX liquid handling robot (Beckman Coulter) was used to dilute the cultures 1/10 into a fresh plate in SC with 3% glycerol. This plate was incubated for 7 hours at 30°C to obtain log phase cells (OD between 0.2 and 0.6 in all cases). An assay plate was then prepared by the Biomek FX in which each well contained 100 *μ*l of log phase cell suspension diluted into a total of 200 *μ*l SC with 3% glycerol (w/v) and 2% glucose (w/v). Fluorescence and optical density data were then recorded using the plate reader.

### Data analysis

A direct approach can be used, which although straightforward does not allow estimates of error and can give negative values of the fluorescence per cell. We estimate the curve *r*_*a*_, which is the ratio of the autofluorescence at 585 nm to that at 525 nm, as a function of OD (Eq. 3) by calculating the mean of *f*585WT/ *f*525WT over all replicates at each time point. To determine the OD at each time point, we average OD values over all replicates and then smooth the resulting average OD curve using local regression with a second degree polynomial (the rloess option in Matlab’s smooth command (Mathworks, Natick, MA)). Each time point therefore corresponds to one average OD value of the wild-type strain. To find the fluorescence of the tagged strain, we find the value of *r*_*a*_ at the corresponding OD, with interpolation when necessary, and then use Eq. 4 to find *g* for each replicate. We average *g* over all replicates and smooth the resulting curve using local regression. Dividing this smoothed curve by the (corrected) OD measurements for the tagged strain gives the mean fluorescence per cell as a function of time.

#### Gaussian processes

We use Gaussian processes to more robustly analyze the data because we can then specify the degree of smoothness of the fits, can make interpolations between and beyond data points, and can straightforwardly propagate errors from one stage of the data analysis to the next.

A Gaussian process is an infinite collection of random variables with each variable associated with a point in some range of an input variable and with any finite number of these random variables having a joint Gaussian distribution [38]. Time is a typical input variable. A Gaussian process is entirely specified by its mean and covariance, both of which are functions of the input variable. When fitting data, we initially consider a Gaussian process with zero mean and with a particular covariance function. The choice of covariance function determines an *a priori* distribution over functions so that samples from the Gaussian process plotted over many input points have characteristic behaviours. For example, if the covariance function is of the neural network form then sampled functions have an *a priori* sigmoid-like shape. If the covariance function is squared exponential, then sampled functions smoothly fluctuate. Given data with measurement noise that is Gaussian, additive, and independently and identically distributed, we can analytically calculate the *a posteriori* mean and covariance function of the Gaussian process by conditioning on the data the joint distribution of both the observed data and the predicted values. By sampling from this *a posteriori* Gaussian process, we can generate functions consistent with the data and our choice of *a priori* covariance function. The variation in these functions is a measure of the error in the fitting. Covariance functions are specified by parameters, called hyperparameters, and we choose the most appropriate hyperparameters by maximizing the marginal likelihood [38] – the probability of the data given the input points – and so by maximizing the evidence for our choice of covariance function. We use a truncated Newton algorithm (from the Python module SciPy) for all optimizations.

#### Correcting the growth curve to be proportional to cell density

To ensure a linear relationship between OD and cell density (the number of cells per unit volume), we used dilution to have an accurate if relative measure of cell density [7]. We diluted a yeast culture (grown overnight on 2% glucose) at a known high OD in a series from 1 × to 256 × in SC media. The cell density of the last sample in the series is therefore 256 times smaller than the cell density of the original sample. By comparing the measured ODs of these samples to their known relative cell densities, we observed that the deviation from linearity begins around an OD of 0.5 (Figure 3c). To correct OD measurements, we would like to find a relative cell density from a measured OD and so performed a regression on the data of Figure 3c. We used a Gaussian process for the regression with a covariance function that is the sum of a squared exponential covariance function and a linear covariance function. Such a covariance function generates smooth functions that increase as the OD increases [38]. For absolute quantification, we used a Neubauer bright-line cytometer (depth: 0.100 mm, grid surface: 0.0025 mm^2^) imaged on a Nikon Eclipse Ti microscope to estimate that a relative cell density of 1.0 for the calibration curve, corresponding to an OD of around 1.2, has an absolute density of 1.46×10^7^ cells/mL.

#### Estimating the mean fluorescence per cell and its error

To find the mean fluorescence per cell and an estimate of its error, we use Gaussian processes. First, we correct the data for the OD of the media in which the cells grow and for its fluorescence. Second, we estimate the curve *r*_*a*_ as a function of OD (Eq. 3) using data from the wild-type strain, and generate sample *r*_*a*_ functions to determine how errors in measuring *r*_*a*_ affect estimates of mean fluorescence. Third, for each time point, we use the measurements of fluorescence at 525 nm and 585 nm for the fluorescently tagged strain, the samples of *r*_*a*_, and samples of the OD correction curve to find a probability distribution of the estimates of the fluorescence per cell. The mean and standard deviation of this distribution are our best-fit value and its error.

##### Correcting for the media

Although we expect both the OD and fluorescence measured in wells containing only media to remain constant over time, both change slightly during the experiment reflecting perhaps small changes in the plate reader. We use a Gaussian process with a squared exponential covariance function to fit data from the media for all replicates. Regression with Gaussian processes typically assumes a constant measurement error but the measurement error in our case changes with time, at least for some of the data sets. We therefore first estimate the measurement errors using the standard deviation of the 10 nearest data points to each data point, and then run a regression with a Gaussian process with measurement errors fixed at these estimated values. If we fail to find hyperparameters by maximizing the marginal likelihood because the optimization algorithm fails to converge, we try an second fit where the measurement error is assumed constant and is itself fit as part of the optimization. Using this combination of methods, we could automatically fit all media data. The OD of the media as a function of time was subtracted from all OD measurements, and the fluorescence of the media as a function of time was subtracted from all fluorescence measurements.

##### Fitting wild-type data to find *r*_*a*_

From Eq. 3, *r*_*a*_ at a particular level of OD is given by the ratio of fluorescence at 585 nm to that at 525 nm for wild-type cells. Figure 3d shows a typical example of this data. We use a Gaussian process with a neural network covariance function, which generates sigmoid-like functions, to find *r*_*a*_. We therefore assume that the measurement error in measuring the ratio of fluorescences has a Gaussian distribution. From Figure 3d, the measurement error changes with OD, and we empirically estimate the measurement error at each OD as the standard deviation of the 20 nearest data points to the data point at that OD. We use this estimated measurement error in our regression (Figure 3d).

We used sampling to estimate how errors in fitting *r*_*a*_ affect the fluorescence per cell. We sampled tens of *r*_*a*_ functions that are consistent with the data and which differ only because of measurement errors, at least given our assumptions. We can therefore determine the error in the estimated fluorescence per cell by finding the standard deviation of the distribution generated by correcting the fluorescence of the tagged strain by each of these sample *r*_*a*_ functions. Although using Gaussian processes make this sampling straightforward, we should sample *r*_*a*_ at the OD levels measured for the tagged strain, not those for the wild-type strain. We can interpolate *r*_*a*_ to these OD values using standard techniques [38], but we must also interpolate the measurement errors to the expected measurement error in *r*_*a*_ at the OD levels of the tagged strain. We use regression with another Gaussian process to fit and interpolate the empirical measurement errors, with a squared exponential covariance function to generate smooth functions. With the interpolated measurement errors and the best-fit hyperparameters of the neural network covariance function found from the wild-type data, we can sample *r*_*a*_ functions.

##### Fitting the data from the tagged strain to find *g*

To find the fluorescence of the tagged strain, *g*, we use a Bayesian approach with uniform prior probabilities so that the posterior probability of *g* is proportional to the likelihood of *g* given the data [39]. From Eq. 1, and, assuming Gaussian measurement errors for measuring each fluorescence value, then the likelihood of *g* at a particular time point is

(5) $$\begin{array}{l} P(\;f_{525},f_{585}|r_{a},r_{g},g,\sigma_{525},\sigma_{585})\\ \;∼\int_{0}^{\infty }\text{da}{\left(\sigma_{585}\sigma_{525}\right)}^{-1}\exp\left[-\frac{{\left(\;f_{525}-g-a\right)}^{2}}{2\sigma_{525}^{2}}\right]\\ \;\times \exp\left[-\frac{{\left(\;f_{585}-r_{g}g-r_{a}a\right)}^{2}}{2\sigma_{585}^{2}}\right]\\ \;∼{\left(\sigma_{585}^{2}\;+\;r_{a}^{2}\sigma_{525}^{2}\right)}^{-\frac{1}{2}}\;\exp\;\left[-\frac{{\left(\;f_{585}\;-\;r_{a}f_{525}\;-\;(r_{g}-r_{a})g\right)}^{2}}{2(\sigma_{585}^{2}+r_{a}^{2}\sigma_{525}^{2})}\right] \end{array}$$

where *σ*_525_ is the measurement error at 525 nm and *σ*_585_ is the measurement error at 585 nm. In Eq. 5, we have extended the range of integration of the autofluorescence, *a*, to -*∞* to *∞* to perform the integration, which should give little effect if the posterior distribution of *a* is sufficiently peaked at a positive value of *a*[39].

We wish to determine both the best-fit value of *g* and its error. We do so by generating a distribution of samples of *g* consistent with the data and take the mean value of *g* as the best estimate and its standard deviation as the error in this estimate. We sample *g* from Eq. 5. For each replicate, we generate 50 samples of *r*_*a*_ functions at the OD levels of the tagged strain. Using the one-to-one relationship between OD and time (Figure 1a), we map the *r*_*a*_ values to the values expected at particular time points. Given a sample of a *r*_*a*_ function, we use rejection sampling to draw 1000 samples of *g* from Eq. 5 [40]. The measurement errors *σ*_525_ and *σ*_585_ are found empirically by taking the standard deviation of the 20 fluorescence measurements closest to the fluorescent measurement at the time point of interest. We include the errors in estimating the relative cell density by sampling a corrected OD function for each sample of *r*_*a*_ and dividing the samples of *g* by this corrected OD. Finally, averaging over 50 (samples of *r*_*a*_ and of corrected OD levels) × 1000 (samples of *g*) × the number of replicates, we find the mean and standard deviation of *g* at each time point.

### Access to data

We have written code to implement our correction method that uses Python and other open source software (available with our raw data at http://swainlab.bio.ed.ac.uk/software/platereader and as Additional file 1).

## Competing interests

The authors declare that they have no competing interests.

## Authors’ contributions

CAL and IBNC developed the experimental method; CAL and PSS wrote the analysis routines; IBNC and RW gathered the data for Figures 1 and 2; CAL and PSS wrote the paper. All authors read and approved the final manuscript.

## Supplementary Material

Additional file 1 **Python software that implements our correction methodology and the data from Figures **1** and **2**.** Click here for file
