# Supplementary material for: Unmixing of fluorescence spectra to resolve quantitative time-series measurements of gene expression in plate readers
Source: BMC Biotechnol. 2014 Feb 3;14:11. doi: 10.1186/1472-6750-14-11 (PMC3917901; doi:10.1186/1472-6750-14-11)
Supplement: Additional file 1 — Python software that implements our correction methodology and the data from Figures 1 and 2. [file 1472-6750-14-11-S1.zip › README.pdf]

Spectral unmixing to resolve quantitative time-series measurements of gene expression in plate readers

Python code and example data: platereadercode.zip (Right click or left click and use the `File' menu to download. Scientific Python is free (www.scipy.org).

Contents

Example: Following expression from the GAL genes
The data structure
Exporting to a text file
Processing using a script
Processing with no annotation file
Correcting OD

Example: Following expression from the GAL genes

We will use the plate reader data shown in Fig. 1 of the paper to demonstrate the analysis. This experiment followed expression of the GAL genes in budding yeast over time and in different sugars.

1. Download, unzip and place the contents of the zipped directory all in one directory. Install Python. This free installation includes NumPy, SciPy, Matplotlib, and IPython. All of which we will use.

2. Start IPython. To start in a Mac, type `ipython --pylab` in a terminal window; to start in Windows, there is usually an icon, such as PyLab for Enthought or A for Anaconda. An IPython window should open.

3. Import the code for analyzing data from plate readers:

   ```
   import platereader as pr
   ```

4. Import the data into Python. The data are assumed to be a tab delimited text file, which Excel can generate as an export option (`.csv` files are also supported). The annotation file is a text file containing a tab delimited table that describes the contents of the wells. For this experiment, the set up was:

water 2% raf	media	WT	WT	GAL1	GAL2	GAL2	GAL3	GAL3	GAL7	GAL10	GAL80	GAL80
water	media	WT	WT	GAL1	GAL2	GAL2	GAL3	GAL3	GAL7	GAL10	GAL80	GAL80

water 1% gal	media	WT	WT	GAL1	GAL2	GAL2	GAL3	GAL3	GAL7	GAL10	GAL80	GAL80
water	media	WT	WT	GAL1	GAL2	GAL2	GAL3	GAL3	GAL7	GAL10	GAL80	GAL80
water 0.1% gal	media	WT	WT	GAL1	GAL2	GAL2	GAL3	GAL3	GAL7	GAL10	GAL80	GAL80
water	media	WT	WT	contaminated	GAL2	GAL2	GAL3	GAL3	GAL7	GAL10	GAL80	GAL80
water 0.01% gal	contaminated	WT	WT	GAL1	GAL2	GAL2	GAL3	GAL3	GAL7	GAL10	GAL80	GAL80
water	media	WT	WT	GAL1	GAL2	GAL2	GAL3	GAL3	GAL7	GAL10	GAL80	GAL80

The description of the media in the first well applies for all rows until a new description is given in the first well of a subsequent row. This description of the media must be separated by a space from the description of the contents of the well. For example, the first well in the first row contains only water and the second well, and as well as all subsequent wells in that row, contains 2% raffinose. Alternatively, the media can be specified in each well. Here wells in the first two rows contain 2% raffinose; the next two rows all have wells with 1% galactose, and so on. Wild-type cells are needed for correcting autofluorescence and are denoted WT. Wells containing only media are also used for the corrections. Wells that are contaminated or empty can be marked so. Here some of the wells contained water but these wells are not used in our correction procedure. All other wells contain strains with the indicated gene fused to GFP.

To import the data, type

```
S= pr.processoriginaldata (fname= 'GALdata.txt', aname=
'GALannotation .txt')
```

which creates a data structure S in Python. Note that both the data and the annotation must be stored in the same type of file (`.txt` files here).

5. We next need to correct for the media. Running

   ```
   pr.mediacorrect (S, figs= 1)
   ```

   performs this correction and displays fits to the data from the wells containing media. Removing the `figs= 1` option will stop these figures from being plotted. This command will take around 10 minutes to run.

6. To process the wild-type data to correct tagged strains for autofluoresence, use

```
pr.processfluorescence(S, noWTattempts= 3)
```

which processes the wild-type data. Here we have specified the variable `noWTattempts= 3`, which is the number of optimization runs used to fit the wild-type data. If this variable is not specified, the default value of 30 is used. Using `noWTattempts= 3`, this code takes around 15 minutes to run.

7. Finally, we correct the tagged strains for autofluoresence:

```
pr.processfluorescence(S, norsamples= 5)
```

which processes all tagged strains. Here we have specified the variable `norsamples= 5`, which is the number of samples of the ratio of autofluorescence curve (Fig. 3d) used to estimate error bars on the corrected values of the fluorescence. If this variable is not specified, the default value of 50 is used. Using `norsamples= 5`, this code takes around 15 minutes to run.

8. To plot, the corrected data use:

```
pr.plotcorrected(S)
```

to graph the corrected fluorescence for all strains. For a particular strain in a particular condition, use, for example:

```
pr.plotcorrected(S, '1%gal', 'GAL7')
```

To plot, the OD curves use:

```
pr.plotraw(S, 'od')
```

or

```
pr.plotraw(S, 'odcorr')
```

to include the corrections for non-linearities between OD and cell density.

The data structure

Running these commands creates the data structure S, which is a Python dictionary. You can access, for example, the data for the GAL10 strain in 1% galactose as `S['1%gal']['GAL10']` with:

`S['1%gal']['GAL10']['t']`	giving the times of measurements in hours
`S['1%gal']['GAL10']['mg']`	giving the corrected fluorescence
`S['1%gal']['GAL10']['sg']`	giving the error on the corrected fluorescence
`S['1%gal']['GAL10']['odata']`	giving the original data
`S['1%gal']['GAL10']['data']`	giving the data corrected for media effects.

For `S['1%gal']['GAL10']['data']`, the first column is OD; the second column is fluorescence measured at 525nm; the third column is fluorescence measured at 585nm. Any replicates are given as three additional columns.

Exporting to a text file

Data can also be exported into a tab delimited text file. For example,

```
pr.export(S, 'corrfluor', '1%gal')
```

will create a file `corrfluor_1%gal.txt` containing the corrected fluorescence and error bars for all genes for all times for cells grown in 1% galactose. If the experimental condition is not specified, a separate file will be created for all conditions measured.

Processing using a script

All the data processing commands can be run in one command using a script, such as `processGAL.py`, by typing

```
%run processGAL.py
```

in IPython or

```
python processGAL.py
```

in a terminal. For tens of genes, the script may require several hours to run.

Processing with no annotation file

If no annotation file is given, then the media is taken to be unknown and the data structure contains information for each well. After running, for example,

```
S= pr.processoriginaldata(fname= 'data.txt')
```

then

```
S['unknown']['A1']['data']
```

returns a two dimensional array for the first well. As before, the first column is OD; the second column is fluorescence measured at 525nm; and the third column is fluorescence measured at 585nm. As before, `pr.plotraw` can used to plot this data.

Correcting OD

We have included here some measurements of OD that allow non-linearities between OD and cell density to be corrected. These measurements are in `dilutions.txt` (the first column is measured OD; the second column is the known dilution factor) and are processed using

```
pr.ODcorrection(fname= 'dilutions.txt')
```

The results of this processing can be stored in a Python `pickle` file by specifing oname when calling the `ODcorrection` function. We have used

`oname= 'corrODparams '`, which creates the `corrODparams .pkl` file already included in the download. This file is used by the `processfluorescence` function and can be specified with the `pfileODparams` variable when calling this function.
